# Network Derivation of Liquid Junction Potentials in Single-Membrane System

**DOI:** 10.3390/membranes14060140

**Published:** 2024-06-13

**Authors:** Andrzej Ślęzak, Sławomir M. Grzegorczyn

**Affiliations:** 1 Collegium Medicum, Jan Dlugosz University, 13/15 Armia Krajowa Al., 42200 Częstochowa, Poland; 2 Department of Biophysics, Faculty of Medical Sciences in Zabrze, Medical University of Silesia, 19 H. Jordan Str., 41808 Zabrze, Poland

**Keywords:** membrane transport, Kedem–Katchalsky–Peusner equations, polymeric membrane, Peusner transport coefficients, internal energy conversion, *S* entropy

## Abstract

Peusner’s network thermodynamics (PNT) is one of the more important formalisms of nonequilibrium thermodynamics used to describe membrane transport and the conversion of the internal energy of the system into energy dissipated in the environment and free energy used for the work involved in the transport of solution components in membrane processes. A procedure of transformation the Kedem–Katchalsky (K-K) equations for the transport of binary electrolytic solutions through a membrane to the Kedem–Katchalsky–Peusner (K-K-P) equations based on the PNT formalism for liquid junction potentials was developed. The subject of the study was a membrane used for hemodialysis (Ultra Flo 145 Dialyser) and aqueous NaCl solutions. The research method was the *L* version of the K-K-P formalism for binary electrolyte solutions. The Peusner coefficients obtained from the transformations of the K-K formalism coefficients for the transport of electrolyte solutions through the artificial polymer membrane were used to calculate the coupling coefficients of the membrane processes and to calculate the dissipative energy flux. In addition, the dissipative energy flux, as a function of thermodynamic forces, made it possible to investigate the energy conversion of transport processes in the membrane system.

## 1. Introduction

Membrane transport is one of the fundamental nonequilibrium processes occurring in various types of physicochemical systems containing biological membranes or artificial polymeric membranes [1]. The study of membrane transport processes provides data of a cognitive and utilitarian nature for many areas of innovative human activities, such as industrial or biomedical technologies. Fuel cells, water and wastewater treatment systems, electric cells, controlled drug release systems, membrane dressings to promote healing of chronic wounds and bioreactors to test strategies to combat bacterial infections using lytic phages in combination with established and novel antimicrobial agents are representative examples of these applications [2,3,4]. In these systems, the appropriate membrane is a selective barrier, ensuring its functionality and effectiveness. This role is fulfilled by, among others, polymeric membranes of different structures and compositions made of polyvinyl chloride, bacterial cellulose or cellulose acetate [5,6].

One of the physical quantities characterizing nonequilibrium systems is thermodynamic entropy (S-entropy) [7]. It numerically characterizes the degree of irreversibility of physicochemical processes subject to the entropy growth law. That is, entropy is a measure of the amount of unused energy in a system. *S*-entropy production is a measure of the irreversibility of mass, charge, energy and momentum transport processes in various types of systems, including physicochemical systems such as, among others, membranes. In turn, the product of entropy production and absolute temperature is a measure of energy dispersion. The energy dissipation function is the starting point for deriving membrane transport equations, as well as equations for internal energy conversion [8]. One of the most popular formalisms for describing membrane transport is the Kedem–Katchalsky equations [9]. They appeared in science at the end of the 1950s and have found applications in many fields of science, technology and biomedicine. In the following years, formalisms and research tools developed within the framework of network thermodynamics appeared.

The first attempts to formulate the principles of network thermodynamics (NT) appeared in the 1960s thanks to the ponderous ideas of Paynter and Meixner. The former developed the bond graph method [10], and the latter noted the relationship between irreversible transport systems and electrical networks [11]. The early 1970s saw the publication of Peusner’s works [12] and Oster, Perelson and Katchalsky’s works [13], which are the pillars of NT. In its modern form, NT is a synthesis of classical nonequilibrium thermodynamics, electrical circuit theory, graph theory and differential geometry [14,15,16]. Its practical application to the analysis of membrane transport is based on Peusner’s idea of NT using nonequilibrium thermodynamics and the symbolism of an analog theory of electric circuits (Kirchhoff’s current and voltage law, Tellegen’s principle, etc.) [14,15,16,17,18], as well as Oster, Perelson and Katchalsky’s idea of NT [13] using Paynter’s bond graph method [1]. Some developments of *L* versions of Peusner’s NT are included in the work of Ślęzak et al. [17,18].

**Figure 1:**
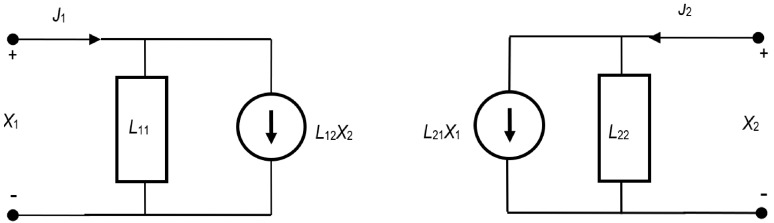
Two-port L representation of the phenomenological equations in which forces ($X_{1},\;X_{2}$) controlling flow sources ($J_{1},\;J_{2}$) are placed in parallel with conductances ($L_{11}$ and $L_{22}$) [14,16].

Following Peusner’s idea and electrical circuit theory, the transducers are labelled L, R, H and P [14,15,16]. Each consists of two dissipative elements (conductance or resistance) and two controllable sources (force, flow or mixed). In the present work, we only deal with the versions of the equations containing the coefficient with the *L* label. Figure 1 shows the L representation of the phenomenological equations in which the force-controlled sources are placed in parallel with the conductance.

A linear two-port representation is described by *L*-version phenomenological equations with two independent variables and two dependent variables. One of the simplest sets of equations is the ‘conductive’ formulation, which is expressed as follows:

(1) $$J_{1}=L_{11}X_{1}+L_{12}X_{2}$$

(2) $$J_{2}=L_{21}X_{1}+L_{22}X_{2}$$

in which the reciprocity relationship is not assumed to be fulfilled [14,15,16]. The notation of Equations (1) and (2) is the same as the Onsager notation. While Equations (1) and (2) are not reciprocal, they share with Onsager thermodynamics that pairs ($J_{1}$, $X_{1}$) and ($J_{2}$, $X_{2}$) are coupled. It should be noted that Lars Onsager did not provide a rigorous phenomenological proof of his reciprocity theorem within the linear thermodynamics of irreversible processes [19]. More than half a century later, Mamedov [20] showed that the reciprocity of the cross-kinetic coefficients is satisfied only when the generalized flows are zero and the generalized forces are unequal to zero, i.e., in fact, at equilibrium. This significantly reduces the value of the reciprocity hypothesis. Within the framework of linear thermodynamics of irreversible processes in the case of a cellular model of an ion-exchange membrane, Filippov showed that the equality of reciprocity coefficients does not apply [21]. The Onsager reciprocity relation of coefficients $L_{12}$ and $L_{21}\;$is also not satisfied under concentration polarization conditions [17].

Network thermodynamics represents these equations using linear conductivities, force sources and flow sources coupled according to Kirchhoff’s laws. One of the main results is that L Equations (1) and (2) can be rearranged, preserving the coupled nature of the forces and flows.

The schemes of circuits shown in Figure 1 are representations of a two-port flow with conductance $G=L_{11}$ connected in parallel to source ${J_{1}}^{\prime }=L_{12}X_{2}$ and conductance $G=L_{22}$ connected to source ${J_{2}}^{\prime }=L_{21}X_{1}$. The total flow in case is represented by Equation (1) and in case (b) by Equation (2).

A previous paper presented procedures for the analysis of membrane transport of binary and ternary homogeneous and heterogeneous non-electrolyte solutions using the L version of the Kedem–Katchalsky–Peusner equations [17,18]. A procedure for the conversion of chemical energy to free energy was also presented in [8]. The first step of this procedure is to calculate the dissipation function using the *L* version of the K-K-P equations. Thermodynamic forces (hydrostatic and osmotic pressure differences), volume and solute fluxes were used in these procedures.

The purpose of this paper is to develop a procedure for converting the K-K equations for binary electrolyte solutions into K-K-P equations using a formalism developed in the context of PNT. Using the obtained *L* version of the K-K-P equations, a method is elaborated to evaluate the conversion of internal energy to free energy in a membrane system containing aqueous electrolyte solutions with a concentration field and an electric field superimposed on them. The work is organized as follows.

The paper begins with a section entitled Introduction. The second section presents the procedure for deriving the *L* versions of the K-K-P equations describing the membrane transport of homogeneous electrolyte solutions and the equations representing the *L* versions of the transport parameters ($L_{ij}$) of the coupling coefficients ($l_{ij}$ and $({Q_{L})}_{ij}$) and the energy conversion efficiency coefficient ([(${{e_{ij})}_{L}]}_{max}$, where (i, j ∊ {1, 2})). In this section, we also present the mathematical equations for the S-energy dissipation function (${\left(\Phi_{S}\right)}_{L}$) derived from the K-K-P formalism describing the energy dissipation function as a function of thermodynamic forces. The derived equations are used to calculate ${\left(\Phi_{S}\right)}_{L}$ = *f*(${∆\pi }_{s}/C_{s}$, *E*), [${{\left(\Phi_{F}\right)}_{L}]}_{ij}$ = *f*(${∆\pi }_{s}/C_{s}$, *E*) and [${{\left(\Phi_{U}\right)}_{L}]}_{ij}$ = *f*(${∆\pi }_{s}/C_{s}$, *E*) based on the ${\left(L\right)}_{ij}$ = *f*(${∆\pi }_{s}/C_{s}$, *E*) and ${\left(L\right)}_{ij}$ = *f*(${∆\pi }_{s}/C_{s}$, *E*) characteristics for an Ultra Flo 145 Dialyzer membrane and aqueous NaCl solutions. The values of the coupling parameter and energy conversion efficiency coefficient [(${{e_{ij})}_{L}]}_{max}$ are used to evaluate electrochemical energy conversion. The third section contains the calculation results and a discussion thereof, and the fourth section contains a summary and conclusions.

## 2. Materials and Methods

### 2.1. Membrane System

**Figure 2:**
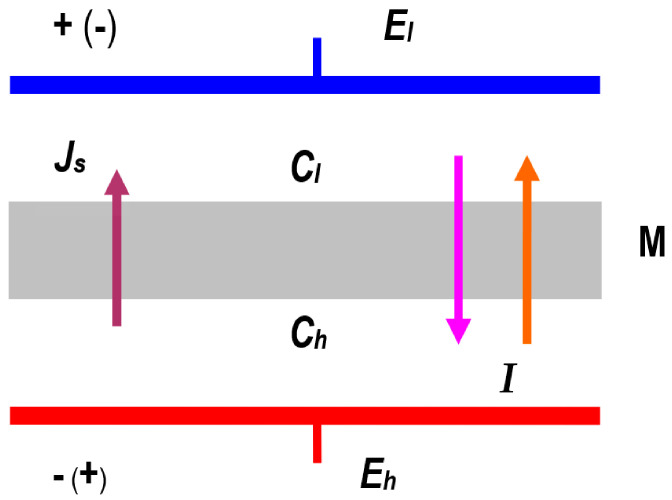
Model of a single-membrane system: M—membrane; $C_{h}$ and $C_{l}$—NaCl solution concentrations ($C_{h}$ > $C_{l}$); $J_{s}$—solute flux; I—electric ionic current; $E_{h}$ and $E_{l}$—electrode potentials.

The system used to study membrane transport is illustrated schematically in Figure 2. This system consists of a membrane (M) located in the horizontal plane and separating two aqueous solutions of NaCl with concentrations at initial moments of $C_{h}$ and $C_{l}$ = const. ($C_{h}$ > $C_{l}$). The density of solutions with concentrations of $C_{h}$ and $C_{l}$ fulfilled the condition $\rho_{h}$ > $\rho_{l}$ = constant. In this system, there are two driving forces, namely $∆\pi =RT(C_{h}-C_{l})$ and $E=(E_{h}-E_{l}$), generating two fluxes ($J_{s}$, I). Identical Ag/AgCl electrodes were placed in $C_{l}$ and $C_{h}$ solutions, between which a voltage of $E=E_{h}-E_{l}$ or $E=E_{l}-E_{h}$ was applied.

According to the Kedem–Katchalsky formalism, the transport parameters of a membrane are determined by the following six coefficients: hydraulic permeability ($L_{p}$), reflection ($\sigma_{s}$), diffusion permeability ($\omega_{s}$), electroosmotic permeability (β), transference number ($\tau_{c}$) and conductance (κ).

### 2.2. L Version of the Kedem–Katchalsky–Peusner Equations

The L versions of the Kedem–Katchalsky–Peusner equations for homogeneous electrolyte solutions are obtained by appropriate transformation of the classical Kedem–Katchalsky equations for homogeneous electrolyte solutions and $J_{v}$ = 0 [15].

(3) $$J_{s}={C_{s}\omega }_{s}\frac{{∆\pi }_{s}}{C_{s}}+\frac{\tau_{c}}{z_{c}F}I$$

(4) $$I=\frac{\kappa \tau_{c}}{z_{c}F}\frac{{∆\pi }_{s}}{C_{s}}+\kappa E$$

where $\omega_{s}$ is the coefficient of diffusion permeability, $J_{s}$ is the solute flux, *I* is the electric ionic current, $∆\pi_{s}$ = RT∆C represents the osmotic pressure difference, RT is the product of the gas constant and the absolute temperature, ∆C = $C_{h}-C_{l}$ ($C_{h}>C_{l}$) represents the difference of concentrations on the membrane, $C_{s}=(C_{h}-C_{l}){\left(lnC_{h}{C_{l}}^{-1}\right)}^{-1}$ = $∆\pi_{s}{\left(RTlnC_{h}{C_{l}}^{-1}\right)}^{-1}$ ≈ 0.5${(C}_{h}+C_{l})$ corresponds to the average concentration of the solution in the membrane, E is the potential difference (voltage) through the membrane, $\tau_{c}$ is the transference number, F is the Faraday constant and κ is the conductance coefficient.

The phenomenological coefficients appearing in Equations (3) and (4) are defined by the following expressions:

(5) $$\omega_{s}={\left(\frac{J_{s}}{{∆\pi }_{s}}\right)}_{\;I=0}$$

(6) $$\kappa ={\left(\frac{I}{E}\right)}_{\;\frac{{∆\pi }_{s}}{C_{s}}=0}$$

(7) $$\tau_{c}=z_{c}F{\left(\frac{J_{s}}{I}\right)}_{\frac{\;{∆\pi }_{s}}{C_{s}}=0\;}$$

To obtain the L version of the Kedem–Katchalsky–Peusner equations for electrolyte solutions, we transform Equations (3) and (4) using Peusner’s network thermodynamics methods. By combining Equations (3) and (4), we obtain the L versions of Equations (3) and (4) as follows:

(8) $$J_{s}=L_{11}\frac{{∆\pi }_{s}}{C_{s}}+L_{12}E$$

(9) $$I=L_{21}\frac{{∆\pi }_{s}}{C_{s}}+L_{22}E$$

where $L_{11}={C_{s}\omega }_{s}+\frac{{\tau_{c}}^{2}\kappa }{F^{2}}$, $L_{12}=\frac{\tau_{c}\kappa }{F}=L_{21}$ and $L_{22}=\kappa$. 

**Figure 3:**
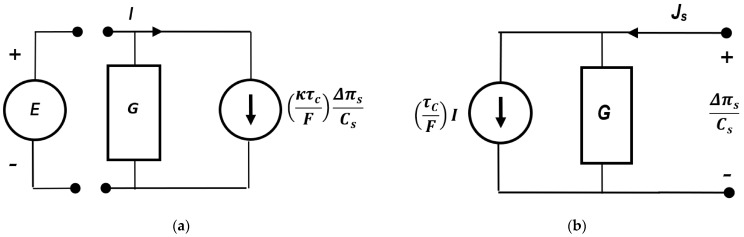
Practical representation of the phenomenological liquid junction potential equations: (**a**) G=κ; (**b**) $G=\omega_{s}C_{s}$.

Equations (8) and (9) can also be written in a matrix form as follows:

(10) $$\left[\begin{array}{c} J_{s}\\ I \end{array}\right]=\left[\begin{array}{cc} L_{11} & L_{12}\\ L_{21} & L_{22} \end{array}\right]\left[\begin{array}{c} \frac{∆\pi }{C_{s}}\\ E \end{array}\right]=[L]\left[\begin{array}{c} \frac{∆\pi }{C_{s}}\\ E \end{array}\right]$$

where $\left[L\right]$ is the matrix of Peusner coefficients ($L_{ij}$ (*i*, *j* ∈ {1, 2})) for binary homogeneous electrolyte solutions. Equations (8)–(10) are among the L forms of the Kedem–Katchalsky equations obtained by means of the symmetrical transformation of Peusner network thermodynamics. A comparison of Equations (8) and (9) shows that for nondiagonal coefficients, $L_{12}$ = $L_{21}$. For fluxes $J_{s}$ and I coupled with forces ${∆\pi }_{s}/C_{s}$ and E, the relations $L_{11}L_{22}$ ≥ ${L_{12}}^{2}$ and $L_{11}L_{22}$ ≥ ${L_{21}}^{2}$ are valid. Furthermore, flux $J_{s}$ can only be coupled with force E if $L_{12}$ ≠ 0. In turn, flux I can only be coupled with force ${∆\pi }_{s}/C_{s}$ if $L_{21}$ ≠ 0. Figure 3 shows the practical representations of phenomenological coupling between fluxes ($J_{s}$, *I*) and thermodynamic forces (${∆\pi }_{s}/C_{s}$, *E*), elaborated on the basis [14].

In turn, we treat the determinant of matrix $\left[L\right]$ as coefficient $det\left[L\right]$ ≡ $L_{det}$

(11) $$L_{det}=C_{s}\omega_{s}\kappa$$

Cross coefficients ($L_{ij(i\neq j)}$) describe the relationship between different irreversible processes. Formed from coefficients $L_{ij}$ (*i*, *j* ∈ {1, 2}), the expression

(12) $$l_{12}=\frac{L_{12}}{\sqrt{L_{11}L_{22}}}={\left(\frac{\kappa {\tau_{c}}^{2}F^{2}}{C_{s}\omega_{s}F^{2}+{\tau_{c}}^{2}\kappa }\right)}^{\frac{1}{2}}$$

determines the degree of coupling between observed processes (Kedem and Caplan coefficients) [22,23]. This means that coefficient $l_{12}$ is a measure of the degree of coupling. If $l_{12}$ = 0, the irreversible processes are independent, while when $l_{12}$ = ±1, the irreversible processes are maximally coupled.

Using Peusner’s definition [14,15,16,17], the energy coupling parameter (Q) can be written in the following form:

(13) $$Q_{L}=\frac{2\left|L_{12}L_{21}\right|}{4L_{11}L_{22}-2L_{12}L_{21}}=\frac{l_{12}l_{21}}{2-l_{12}l_{21}}=Q_{l}=\frac{{{\kappa \tau }_{c}}^{2}}{2C_{s}\omega_{s}F^{2}+\kappa {\tau_{c}}^{2}}$$

This parameter can be used to study the efficiency and stability of physicochemical and biological systems performing energy conversion.

The concept of the degree of coupling is used to determine the energy conversion efficiency $({e_{12})}_{L}$, 0 ≤ $({e_{12})}_{L}$ ≤ 1. The maximum value of this coefficient is determined by the following expression:

(14) $${\left(e_{L}\right)}_{max}=\frac{L_{12}L_{21}}{L_{11}L_{22}{\left(1+\sqrt{1-\frac{L_{12}L_{21}}{L_{11}L_{22}}}\right)}^{2}}=\frac{2Q_{L}}{{(1+Q}_{L}){\left(1+\sqrt{1-\frac{2Q_{L}}{1+Q_{L}}}\right)}^{2}}=\frac{l_{12}l_{21}}{{\left(1+\sqrt{1-l_{12}l_{21}}\right)}^{2}}={\left(e_{l}\right)}_{max}$$

Equation (14) illustrates the relationship between the degree of coupling and the maximum efficiency of energy conversion. It is worth mentioning that full coupling ($l_{12}$ = 1) occurs at $({e_{12})}_{l}$ = 1. This means that the stationary states of flows characterized by minimum entropy production are identical to the state with maximum efficiency.

### 2.3. Mathematical Model of Energy Conversion in the Membrane System

The measure of S-energy dissipation is the so-called dissipation function ($\Phi_{S}$), which is equal to the product of absolute temperature (*T*) and S-entropy production ($d_{i}S/dt$). To obtain mathematical expressions for *S*-energy dissipation in a system in which a membrane separates two homogeneous electrolytic solutions of different concentrations, we use the procedure described in a previous paper [8].

For stationary membrane transport of homogeneous electrolytic solutions containing one solute and a solvent, for conditions of $J_{v}$ = 0, the equation for the L version of the dissipation function takes the following form:

(15) $${\left(\Phi_{S}\right)}_{L}={\left(\Phi_{S}\right)}_{J_{s}}+{\left(\Phi_{S}\right)}_{I}=J_{s}\frac{∆\pi_{s}}{C_{s}}+IE$$

We now calculate the ${\left(\Phi_{S}\right)}_{L}$ of Equation (15) using the L versions of the Kedem–Katchalsky–Peusner equations. Combining Equations (8) and (9) with Equation (15), we obtain the following:

(16) $${\left(\Phi_{S}\right)}_{L}=L_{11}{\left(\frac{∆\pi_{s}}{C_{s}}\right)}^{2}+\left(L_{12}+L_{21}\right)E\frac{∆\pi_{s}}{C_{s}}+L_{22}E^{2}$$

Taking into account in Equation (15), as well as the expressions for $L_{11}$, $L_{12}$ = $L_{21}$ and $L_{22}$ found in Equations (8) and (9), we obtain the following:

(17) $${\left(\Phi_{S}\right)}_{L}=\left({C_{s}\omega }_{s}+\frac{{\tau_{c}}^{2}\kappa }{F^{2}}\right){\left(\frac{∆\pi_{s}}{C_{s}}\right)}^{2}+2\frac{\tau_{c}\kappa }{F}E\frac{∆\pi_{s}}{C_{s}}+\kappa E^{2}$$

In thermodynamic systems, as well as in membrane systems, the internal energy (*U*-energy) can be converted into free energy (*F* energy) and dissipated energy (*S* energy) [8]. The fluxes of these quantities satisfy the following equation:

(18) $${\left(\Phi_{U}\right)}_{L}={\left(\Phi_{F}\right)}_{L}+{\left(\Phi_{S}\right)}_{L}$$

where ${\left(\Phi_{U}\right)}_{L}=A^{-1}dU/dt$ is the flux of *U* energy, ${\left(\Phi_{F}\right)}_{L}=A^{-1}dF/dt$ is the flux of *F* energy, ${\left(\Phi_{S}\right)}_{L}=TA^{-1}\;d_{i}S/dt$ is the flux of dissipated energy (*S*-energy), $d_{i}S/dt$ is the rate of entropy creation by irreversible processes in the membrane system (flux of cumulative entropy production; *T*, absolute temperature; *A*, the membrane surface area. Equations (16) and (17) show the L version of the S-energy dissipation. ${\left(\Phi_{S}\right)}_{L}$ is the flux of dissipated energy, i.e., the time change of energy per unit area of the membrane expressed in W/m^2^. We can calculate the ${\left(\Phi_{F}\right)}_{L}$ and ${\left(\Phi_{U}\right)}_{L}$ for concentration polarization conditions using the following equation [8]:

(19) $${\left(e_{L}\right)}_{max}=\frac{{\left(\Phi_{F}\right)}_{L}}{{\left(\Phi_{U}\right)}_{L}}=\frac{{\left(\Phi_{F}\right)}_{L}}{{\left(\Phi_{F}\right)}_{L}+{\left(\Phi_{S}\right)}_{L}}$$

Transforming Equation (19), we obtain

(20) $${\left(\Phi_{F}\right)}_{L}=\frac{{\left(e_{L}\right)}_{max}}{1-({e_{L})}_{max}}{\left(\Phi_{S}\right)}_{L}$$

(21) $${\left(\Phi_{U}\right)}_{L}=\frac{1}{1-({e_{L})}_{max}}{\left(\Phi_{S}\right)}_{L}$$

where $({e_{L})}_{max}$ is the energy conversion efficiency defined by means of Kedem–Caplan–Peusner coefficients and can be presented in the following form:

(22) $$({e_{L})}_{max}=\frac{{\kappa \tau_{c}}^{2}}{{\kappa \tau_{c}}^{2}+2\left\{C_{s}\omega_{s}F^{2}+{\left[\left(C_{s}\omega_{s}F^{2}+{\kappa \tau_{c}}^{2}\right)C_{s}\omega_{s}F^{2}\right]}^{\frac{1}{2}}\right\}}$$

From a formal point of view, the cases of ${\left(\Phi_{F}\right)}_{L}=0\;$and ${\left(\Phi_{U}\right)}_{L}$ = 0 are excluded because in order for the denominator of Equations (20) and (21) to be different from zero, the condition of $({e_{L})}_{max}$ ≠ 1 must be satisfied.

The values of $({e_{L})}_{max}$ coefficients are limited by the relations 0 ≤ $({e_{L})}_{max}$ ≤ 1; $({e_{L})}_{max}$ = 0 when $L_{12}L_{21}$ = 0 or $l_{12}l_{21}$ = 0 and $({e_{L})}_{max}$ = 1 when $L_{12}L_{21}$ = $L_{11}L_{22}$ or $l_{12}l_{21}$ = 1. The values of the coefficients ($({e_{L})}_{max}$) are limited by the relation 0 ≤ $({e_{L})}_{max}$ ≤ +1. Taking into account Equation (14) in (20), we obtain the following:

(23) $${\left(\Phi_{F}\right)}_{L}=\frac{L_{12}L_{21}}{L_{11}L_{22}{\left(1+\sqrt{1-\frac{L_{12}L_{21}}{L_{11}L_{22}}}\right)}^{2}-L_{12}L_{21}}{\left(\Phi_{S}\right)}_{L}$$

To obtain the equation for ${\left(\Phi_{U}\right)}_{L}$, it is necessary to take into consideration Equation (14) in Equation (21). After performing the necessary transformations, we obtain

(24) $${\left(\Phi_{U}\right)}_{L}=\frac{L_{11}L_{22}L^{2}}{L_{11}L_{22}L^{2}-L_{12}L_{21}}{\left(\Phi_{S}\right)}_{L}$$

where $L=1+\sqrt{1-\frac{L_{12}L_{21}}{L_{11}L_{22}}}$.

Following the above procedure, based on Equations (14)–(24), we can calculate the amount of available *F* energy that can be converted into useful work and the total internal *U* energy.

### 2.4. Biomembrane Characteristics

**Figure 4:**
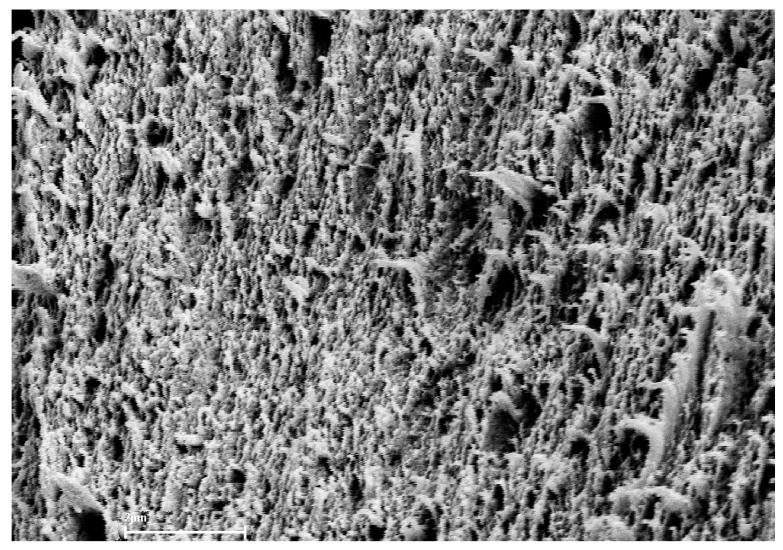
Image of the Ultra Flo 145 Dialyzer membrane obtained by a scanning microscope (Zeiss Supra 35) at 10,000× magnification.

The biomembranes used in the study were Ultra Flo 145 Dialyzer hemodialysis membranes (Artificial Organs Division, Travenol Laboratories S.A., Brussels, Belgium) [24]. The membrane is made of regenerated cellulose and is symmetrical, hydrophilic, isotropic and electroneutral. The scanning image of the Ultra Flo 145 Dialyser membrane shown in Figure 4 has a compact structure with visible cellulose fiber residues. This structure gives the membrane its high stiffness and strength. For research purposes, the Ultra Flo 145 Dialyser membrane was cut in the form of a disc from a hemodialysis hose that was part of a ‘coiled artificial kidney’ used in medicine in the second half of the 20th century [24].

The membrane was separated into two vessels of equal volumes made of Plexiglas and filled with aqueous NaCl solutions of different concentrations. One of the vessels was connected to a calibrated pipette and the other to a solution reservoir. A Ag/AgCl electrode in the form of a flat disc was placed in each vessel. The electrodes were of equal thickness and had equal surface areas. Voltage was applied to the electrodes using a suitable DC power supply. The experiments were performed in an isolated and grounded metal chamber to ensure constant temperature (*T* = 295 K) and to eliminate the influence of external electrical interferences.

**Figure 5:**
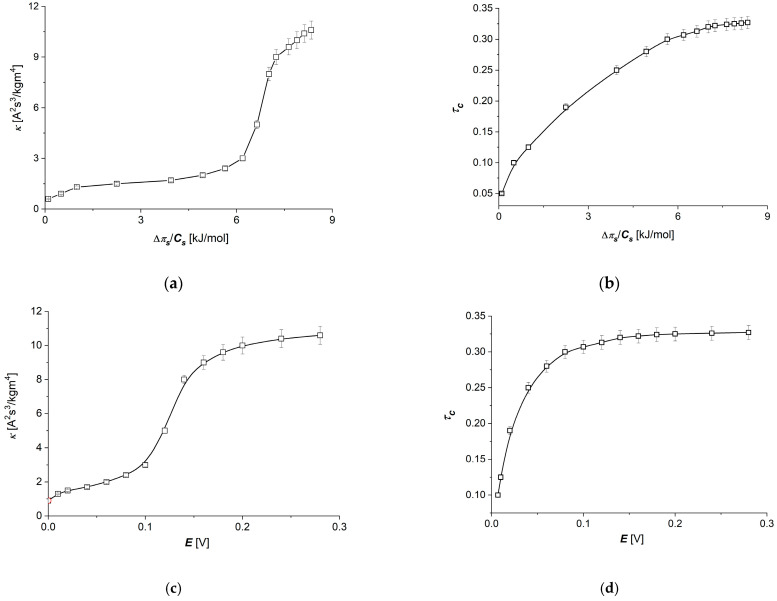
Dependencies $\kappa ={f(∆\pi /C_{s})}_{E=0.15\;V.}$ (**a**), $\tau_{c}={f({∆\pi /C}_{s})}_{E=0.15\;V.}$ (**b**), $\kappa =f{\left(E\right)}_{{∆\pi /C}_{s}=6.64kJ/mol}$ (**c**) and $\tau_{c}=f{\left(E\right)}_{{∆\pi /C}_{s}=6.64kJ/mol}$ (**d**) for Ultra Flo 145 Dialyzer membrane and aqueous NaCl solutions.

The values of transport coefficients ($\omega_{s}$) of the Ultra Flo 145 Dialyser membrane appearing in Equations (1) and (2) in the studied range of NaCl concentrations are constant, and $\omega_{s}$ = 5.5 × 10^−10^ mol/Ns. In turn, the values of transport coefficients κ and $\tau_{c}$ in the studied range of NaCl concentrations are concentration-dependent. The dependencies $\kappa =f(∆\pi /C_{s},\;E=0.15\;V)$ and $\tau_{c}=f({∆\pi /C}_{s},\;E=0.15\;V)$ are presented in Figure 5a,b, while Figure 5c,d show dependencies $\kappa =f(E,\;{∆\pi /C}_{s}$ = 6.64 kJ/mol) and $\tau_{c}=f(E,\;{∆\pi /C}_{s}$ = 6.64 kJ/mol). The abovementioned relations were determined according to the procedure described in the [25]. The dependencies of relations $\kappa =f(∆\pi /C_{s}$, *E*$=0.15)\;and\;\tau_{c}=f\left({∆\pi }_{s}/C_{s},\;E=0.15\;V\right)$ in Figure 5a,b and $\kappa =f(E,{∆\pi /C}_{s}$ =6.64 kJ/mol) and $\tau_{c}=$ $f(E,{∆\pi /C}_{s}$ =6.64 kJ/mol) in Figure 5c,d are nonlinear curves and increase with increases in ${∆\pi }_{s}/C_{s}$ or E.

The change of direction to ${∆\pi }_{s}/C_{s}$ or E on the membrane yields the same values of parameters κ and $\tau_{c}$ in both directions of thermodynamic fluxes because the Ultra Flo 145 Dialyser membrane is symmetrical due to the direction of transport of substances through the membrane. The values of the coefficients κ and $\tau_{c}$ are positive, irrespective of the sign of E or ${∆\pi }_{s}/C_{s}$.

An increase in ${∆\pi }_{s}/C_{s}$ up to 6 kJ/mol causes a very slow increase in the conductance coefficient (κ) (Figure 5a), followed by a quick, nearly three-fold increase in κ with ${∆\pi }_{s}/C_{s}$ between 6 and 7.5 kJ/mol. In this range of ${∆\pi }_{s}/C_{s}$ (from 0 to 6 kJ/mol), the transference number increases nearly linearly. As shown in Figure 5c, the maximal change in the conductance coefficient (*κ*) is observed for the electrical potential difference on the membrane (E) between 0.1 and 0.2 V and for transference number (*τ*_c_) from 0 to 0.1 V (Figure 5d). These dependencies show that the linear model for ion transport through the Ultra Flo 145 Dialyser membrane described by Equations (8) and (9) is valid for the same ranges of changes of thermodynamic forces (${∆\pi }_{s}/C_{s}$ and E), while for an exact description of these processes, a nonlinear model should be used—in our case, as taken into account by the dependence of the model’s transport coefficients (κ and$\;\tau_{c}$) on thermodynamic forces.

The conductance coefficient for the membrane (*κ*) and the transference number ($\tau_{c}$), as parameters of the electrical transport properties through the membrane, play an important role in ion transport not only through artificial membranes but also biological membranes. Their dependence on the thermodynamic force used for the Ultra Flo 145 Dialyser membrane indicates nonlinear dependencies of thermodynamic fluxes (current densities and ion flux) on the thermodynamic forces (differences in voltages or concentrations on the membrane). As Figure 5 shows, in the range of lower values of thermodynamic forces (up to 0.1 V or up to approximately 6 kJ/mol), the conductance coefficient of the Ultra Flo 145 Dialyser membrane changes only to a small extent, which, with some approximation, could be considered in accordance with the model of linear thermodynamics for the range of linear relationship between thermodynamic forces and fluxes. For higher values of thermodynamic forces, the changes in the conductance coefficient of the membrane with changes in the thermodynamic force are much greater. The conductance coefficient, which is related to the density of ions transported through the membrane, as a coefficient characterizing the membrane, can be defined as consistent with linear thermodynamics for small values of thermodynamic forces on the membrane, with an increasing divergence from linear relationships with increasing thermodynamic forces on the membrane. The conclusion that can be drawn from this is that increasing the thermodynamic force on the membrane causes an increase in the ion flux through the membrane. However, for high values of thermodynamic forces on the membrane, a slowing increase in the conductance coefficient is observed, which may indicate the observed tendencies of the onset of the “saturation effect” related to the limited capabilities of the membrane to transport ions at high values of thermodynamic forces (this is especially visible for an electrical force). As for the transference number, in this case, for an electrical force, large changes are observed in the range of small electrical force values (up to 0.08 V), while for larger thermodynamic forces (greater than 0.14 V), the transference number practically does not change. For a concentration difference on the membrane, almost in the entire range of tested concentrations, the transference number increases with increasing concentration difference on the membrane. This indicates a strong dependence of ion transport on the thermodynamic force on the membrane, even for small values of concentration difference. Also in this case, for higher values of thermodynamic forces on the membrane, the beginning of the “saturation effect” can be observed for slightly lower values than in the case of the conductance coefficient of the membrane. The behavior of these two easily measurable electrical parameters of the membrane, which are the starting point for obtaining subsequent parameters in the presented model, in addition to providing broader descriptions of the phenomena of electrolyte transport through the membrane, significantly influence the nature of the obtained relationships.

## 3. Results and Discussion

### 3.1. The Characteristics $L_{ij}=f(E,$ ${∆\pi }_{s}/C_{s}$) (i, j ∈ {1, 2}) and $L_{det}=f(E,$ ${∆\pi }_{s}/C_{s}$) 

**Figure 6:**
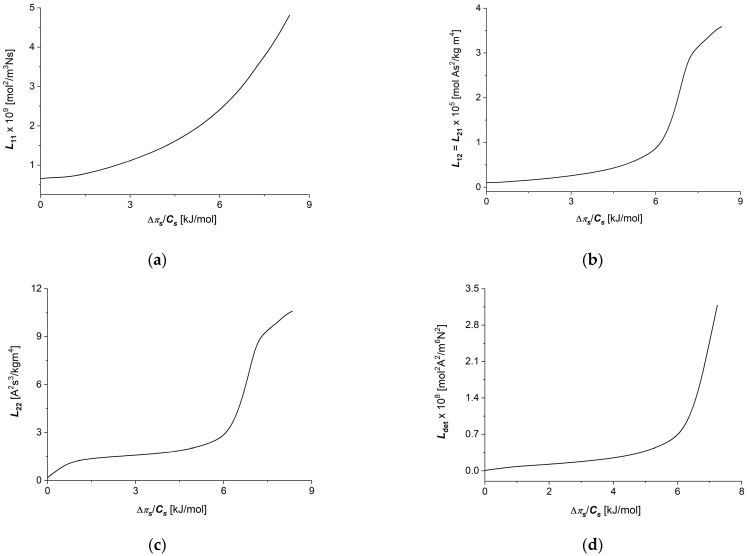
Graphic illustration of dependencies $L_{ij}={f({∆\pi }_{s}/C_{s})}_{E=0.15\;V}$ (i,j ∈ {1, 2}) and $L_{det}={f({∆\pi }_{s}/C_{s})}_{E=0.15\;V}$ for aqueous NaCl solutions: (**a**) $L_{11}={f({∆\pi }_{s}/C_{s})}_{E=0.15\;V}$; (**b**) $L_{12}=L_{21}={f({∆\pi }_{s}/C_{s})}_{E=0.15\;V}$; (**c**) $L_{22}={f({∆\pi }_{s}/C_{s})}_{E=0.15\;V}$; (**d**) $L_{det}={f({∆\pi }_{s}/C_{s})}_{E=0.15\;V}$.

Calculations of the coefficients $L_{ij}=f({∆\pi }_{s}/C_{s})$ (i, j ∈ {1, 2}) and $L_{det}=f\left({∆\pi }_{s}/C_{s}\right)$ (for constant E = 0.15 V) were performed for the following data: R = 8.31 J/mol K, T = 295 K, F = 9.65 × 10^4^ C/mol, $C_{l}$ = 1 mol/m^3^, $C_{h}$ ∈ {1 ÷ 20 mol/m^3^} and ${∆\pi }_{s}/C_{s}$ ∈ {0 ÷ ±7.25 kJ/mol}. To calculate the dependencies $L_{ij}=f({∆\pi }_{s}/C_{s},\;E=0.15\;V)$, (i, j ∈ {1, 2}) and $L_{det}=f\left({∆\pi }_{s}/C_{s},\;E=0.15\;V\right)$, Equations (6)–(9) were used. The results of the calculations are presented in Figure 6a–d. It can be seen from these figures that the graphs illustrating dependencies $L_{ij}=f({∆\pi }_{s}/C_{s},\;E=0.15\;V)$, (i, j ∈ {1, 2}) and $L_{det}=f\left({∆\pi }_{s}/C_{s},\;E=0.15\;V\right)$ are nonlinearly increasing functions of ${∆\pi }_{s}/C_{s}$. Changing the sign of ${∆\pi }_{s}/C_{s}$ from positive to negative (oppositely directed ${∆\pi }_{s}/C_{s}$ on the membrane) does not change either the value or sign of $L_{ij}=f\left({∆\pi }_{s}/C_{s},\;E=0.15\;V\right)$ (i, j ∈ {1, 2}) or $L_{det}=f\left({∆\pi }_{s}/C_{s},\;E=0.15\;V\right)$. Note that $L_{ij}=f\left({∆\pi }_{s}/C_{s},\;E=0.15\;V\right)$, (i, j ∈ {1, 2}) and $L_{det}=f\left({∆\pi }_{s}/C_{s},\;E=0.15\;V\right)$.

**Figure 7:**
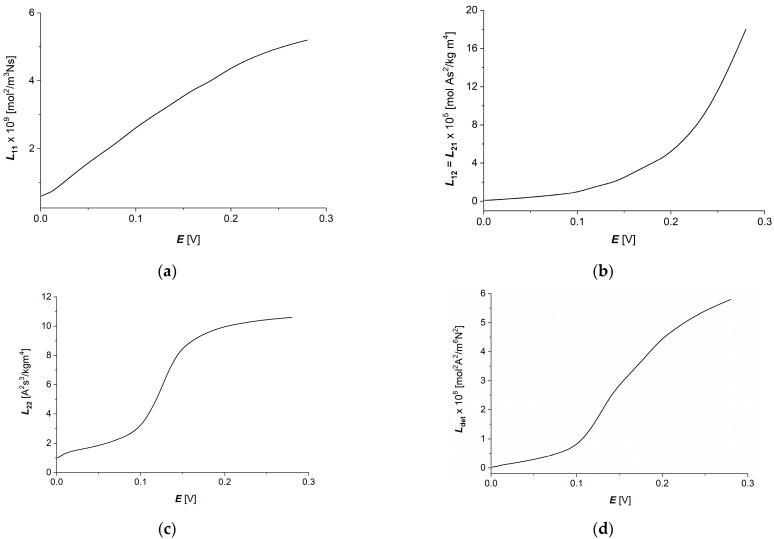
Graphic illustration of dependencies $L_{ij}={f(E}_{∆\pi /C_{s}=6.64kJ/mol}),$ (i,j ∈ {1, 2}) and $L_{det}=f(E_{∆\pi /C_{s}=6.64kJ/mol}),$ for aqueous NaCl solutions: (**a**) $L_{11}=f(E,$ ${∆\pi }_{s}/C_{s}$ = 6.64 kJ/mol), (**b**) $L_{12}=L_{21}=f(E,{∆\pi }_{s}/C_{s}$ = 6.64 kJ/mol) (**c**) $L_{22}=f(E,$ ${∆\pi }_{s}/C_{s}$ = 6.64 kJ/mol) and (**d**) $L_{det}=f(E,$ ${∆\pi }_{s}/C_{s}$ = 6.64 kJ/mol).

Calculations of coefficients $L_{ij}=f(E,$ ${∆\pi }_{s}/C_{s}$ = 6.64 kJ/mol) (i, j ∈ {1, 2}) and $L_{det}=f(E,$ ${∆\pi }_{s}/C_{s}$ = 6.64 kJ/mol) were performed for the following data: R = 8.31 J/(mol K), T = 295 K, F = 9.65 × 10^4^ C/mol and ${∆\pi }_{s}/C_{s}$ = ±6.64 kJ/mol. To calculate dependencies $L_{ij}=f(E,$ ${∆\pi }_{s}/C_{s}$ = 6.64 kJ/mol) (i, j ∈ {1, 2}) and $L_{det}=f(E,$ ${∆\pi }_{s}/C_{s}$ = 6.64 kJ/mol), Equations (8)–(11) were used. The results of the calculations are presented in Figure 7a–d. It can be seen from these figures that the graphs illustrating dependencies $L_{ij}=f(E,$ ${∆\pi }_{s}/C_{s}$ = 6.64 kJ/mol) (i, j ∈ {1, 2}) and $L_{det}=f(E,$ ${∆\pi }_{s}/C_{s}$ = 6.64 kJ/mol) are also nonlinear increasing functions of potential difference on the membrane (*E*).

Changing the sign of the potential difference on the membrane (*E*) from positive to negative does not change either the value or sign of $L_{ij}=f(E,$ ${∆\pi }_{s}/C_{s}$ = 6.64 kJ/mol) (i, j ∈ {1, 2}) or $L_{det}=f(E,$ ${∆\pi }_{s}/C_{s}$ = 6.64 kJ/mol). We can state that the Ultra Flo 145 Dialyzer membrane is symmetrical in relation to the applied potential difference on the membrane.

The coupling coefficients between thermodynamic forces and fluxes ($L_{ij}$) obtained from the measured conductance coefficient (κ), transference number ($\tau_{c}$) and permeability coefficient ($\omega_{s}$) show a strong dependence on the thermodynamic forces. In all cases of $L_{ij}$ coefficients, increasing the thermodynamic forces on the membrane results in a faster increase, as well as increased in the thermodynamic fluxes appropriately coupled to the thermodynamic forces. Also in this case, for some membrane processes (electrical force and current density through the membrane—coefficients $L_{22}$ and $L_{12}$), in the range of small values of each of the thermodynamic forces, small changes in the values of these coupling coefficients can be observed, resulting in increasingly faster changes with the onset of the “saturation effect” for large values of thermodynamic forces. In the case of the coupling process of concentration difference and ion flux through the membrane, characterized by the coupling coefficient ($L_{11}$), analogous changes in the $L_{11}$ coefficient are observed in the entire range of applied concentrations, without a clear transition to the “saturation effect”.

### 3.2. Characteristics $l_{ij}=f({∆\pi }_{s}/C_{s},E)$, (i, j ∈ {1, 2, 3}) and $Q_{L}=f({∆\pi }_{s}/C_{s},E)$

**Figure 8:**
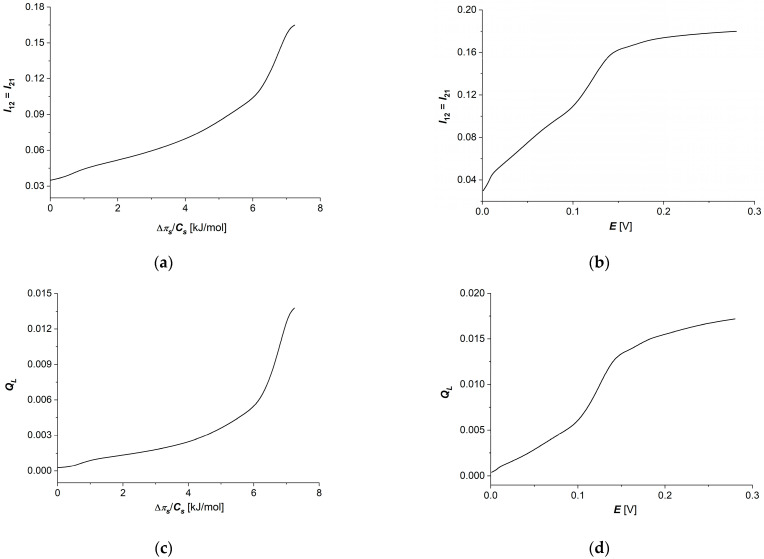
Graphic illustration of dependencies $l_{12}=l_{21}=f({∆\pi }_{s}/C_{s},E=0.15\;V)$ (**a**), $l_{12}=l_{21}=f(E,{∆\pi }_{s}/C_{s}=6.64\;kJ/mol)$ (**b**), $Q_{L}=f({∆\pi }_{s}/C_{s},E=0.15\;V)$ (**c**) and $Q_{L}=f(E,∆\pi /C_{s}=6.64\;kJ/mol)$ (**d**) for aqueous NaCl solutions.

Taking into account the results of calculations obtained for dependencies $L_{ij}=f({∆\pi }_{s}/C_{s},\;E=0.15\;V)$ and $L_{ij}=f(E,$ ${∆\pi }_{s}/C_{s}$ = 6.64 kJ/mol) (i, j ∈ {1, 2}) shown in Figure 6a,c and Figure 7a,c in Equation (12), dependencies $l_{12}=f({∆\pi }_{s}/C_{s},\;E=0.15\;V)$ and $l_{12}=f(E,$ ${∆\pi }_{s}/C_{s}$ = 6.64 kJ/mol) were calculated. The curves presented in Figure 8a,b show that characteristics $l_{12}=f({∆\pi }_{s}/C_{s},\;E=0.15\;V)$ and $l_{12}=f(E,$ ${∆\pi }_{s}/C_{s}$ = 6.64 kJ/mol) are nonlinear, and $l_{12}$ = $l_{21}$. Moreover, these characteristics are symmetrical with respect to the change in direction of thermodynamic forces ${∆\pi }_{s}/C_{s}$ and *E* on the membrane.

Considering the $l_{12}=f({∆\pi }_{s}/C_{s},E=0.15\;V)$ and $l_{12}=f(E,$ ${∆\pi }_{s}/C_{s}$ = 6.64 kJ/mol) dependencies shown in Figure 7a,b and Equation (13), dependencies $Q_{L}=f({∆\pi }_{s}/C_{s})$ and $Q_{L}=f(E,$ ${∆\pi }_{s}/C_{s}$ = 6.64 kJ/mol) were calculated. The curves presented in Figure 8c,d show that characteristics $Q_{L}=f({∆\pi }_{s}/C_{s},E=0.15\;V)$ and $Q_{L}=f(E,$ ${∆\pi }_{s}/C_{s}$ = 6.64 kJ/mol) are nonlinear, increasing the functions of ${∆\pi }_{s}/C_{s}$ or E.

As for coefficients ($l_{ij}$), characterizing the degree of coupling between the observed processes (in this case, cross-couplings such as electrical force–ion flux or concentration stimulus and current density through the membrane) the increase of thermodynamic force on the membrane causes an increase in the degree of coupling of the processes within the following areas: slower growth for small thermodynamic force values, rapid growth for intermediate values of thermodynamic forces and the beginning of the “saturation effect” for larger values of thermodynamic forces. Similar effects (in similar concentration ranges of the relevant thermodynamic forces) can be seen for the energy coupling coefficient of the observed processes ($Q_{L}$), which indicates an increasing energy coupling of the processes with increase thermodynamic forces with the “saturation effect”; especially visible for the electrical force on the membrane. As can be seen, both the degree of coupling between individual processes and the energy coupling coefficient of these processes behave in an “analogous” way.

**Figure 9:**
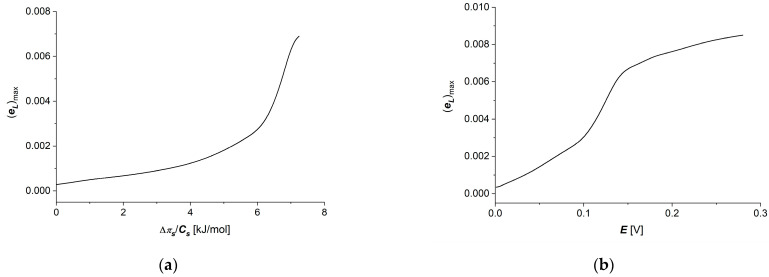
Graphic illustration of dependencies (${e_{L})}_{max}=f({∆\pi }_{s}/C_{s},E=0.15\;V)$ (**a**) and (${e_{L})}_{max}=f(E,$ ${∆\pi }_{s}/C_{s}$ = 6.64 kJ/mol) (**b**) for aqueous NaCl solutions.

Considering the $l_{12}=f({∆\pi }_{s}/C_{s},E=0.15\;V)$ and $l_{12}=f(E,$ ${∆\pi }_{s}/C_{s}$ = 6.64 kJ/mol) dependencies shown in Figure 8a,b and Equation (14), dependencies (${e_{L})}_{max}=f({∆\pi }_{s}/C_{s},E=0.15\;V)$ and (${e_{L})}_{max}=f(E,$ ${∆\pi }_{s}/C_{s}$ = 6.64 kJ/mol) were calculated. These dependencies are presented in Figure 9a,b.

The energy conversion efficiency coefficient of membrane processes reaches values lower than 0.01, and its dependencies on thermodynamic forces are similar to the energy coupling coefficient ($Q_{L}$) dependencies, with a strongly marked “saturation effect” for the electrical force (Figure 9b) and a poorly visible effect for the concentration difference on the membrane (Figure 9a).

### 3.3. Characteristics ${\left[{{(\Phi }_{S})}_{L}\right]}_{E=const}=f({∆\pi }_{s}/C_{s})$ and ${\left[{{(\Phi }_{S})}_{L}\right]}_{{∆\pi }_{s}/C_{s}=const}=f(E)$

**Figure 10:**
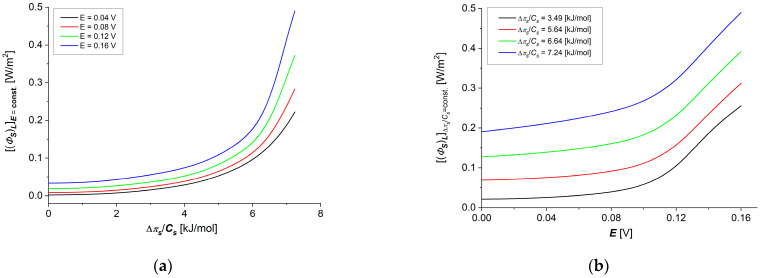
Graphic illustration of dependencies ${\left[{{(\Phi }_{S})}_{L}\right]}_{E=const}=f(∆\pi_{s}/C_{s})$ (**a**) and ${\left[{{(\Phi }_{S})}_{L}\right]}_{{∆\pi }_{s}/C_{s}=const}=f(E)$ (**b**) for aqueous NaCl solutions.

Taking into account the results of the calculations obtained for $L_{ij}=f(∆\pi_{s}/C_{s})$, (i, j ∈ {1, 2}) shown in Figure 6a–d and Figure 7a–d in Equation (16), dependencies ${\left(\Phi_{S}\right)}_{L}=f(E,{∆\pi }_{s}/C_{s}=6.64kJ/mol)\;$and ${\left(\Phi_{S}\right)}_{L}=f(E,$ ${∆\pi }_{s}/C_{s}$ = 6.64 kJ/mol) were calculated. The results of the calculations are presented in Figure 10a,b. The graphs shown in these figures are nonlinear, increasing functions of thermodynamic forces ($∆\pi_{s}/C_{s}$) (Figure 10a) or E (Figure 10b). From Figure 10a,b, it can be seen that ${\left(\Phi_{S}\right)}_{L}$ increases both with an increase in $∆\pi_{s}/C_{s}$ at a fixed value of E and with an increase in E at a fixed value of $∆\pi_{s}/C_{s}$.

### 3.4. Characteristics ${\left[{{(\Phi }_{F})}_{L}\right]}_{E=const}=f({∆\pi }_{s}/C_{s})$, ${\left[{{(\Phi }_{F})}_{L}\right]}_{{∆\pi }_{s}/C_{s}=const}=f(E)$, ${\left[{{(\Phi }_{U})}_{L}\right]}_{E=const}=f({∆\pi }_{s}/C_{s})$ and ${\left[{{(\Phi }_{U})}_{L}\right]}_{{∆\pi }_{s}/C_{s}=const}=f(E)$

**Figure 11:**
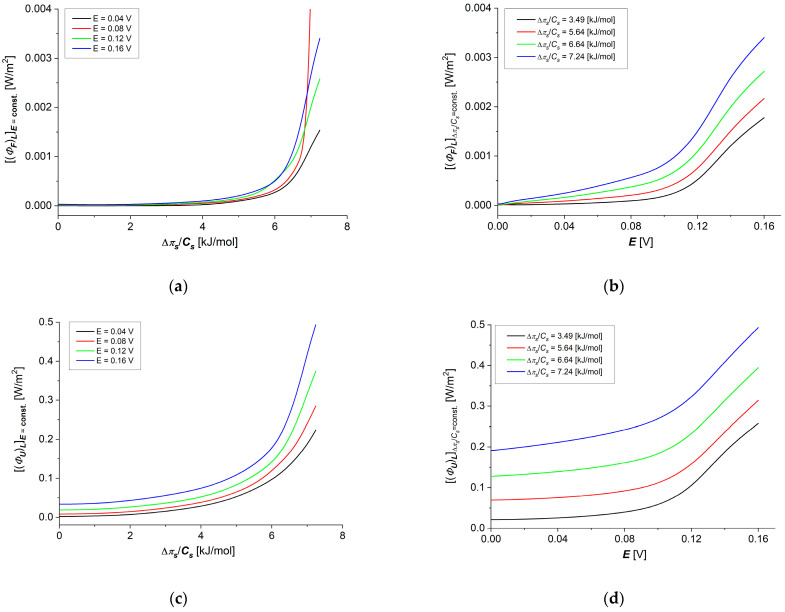
Graphic illustration of dependencies ${\left({\left[{{(\Phi }_{F})}_{L}\right]}_{12}\right)}_{E=const}=f({∆\pi }_{s}/C_{s})$ (**a**), ${\left({\left[{{(\Phi }_{U})}_{L}\right]}_{12}\right)}_{E=const}=f({∆\pi }_{s}/C_{s})$ (**c**), ${\left({\left[{{(\Phi }_{F})}_{L}\right]}_{12}\right)}_{{∆\pi }_{s}/C_{s}=const}=f(E)$ (**b**) and ${\left({\left[{{(\Phi }_{U})}_{L}\right]}_{12}\right)}_{{∆\pi }_{s}/C_{s}=const}=f(E)$ (**d**) for aqueous NaCl solutions.

Taking into account the results of calculations obtained for dependencies (${e_{L})}_{max}=f({∆\pi }_{s}/C_{s})$ shown in Figure 9a,b in Equations (22) and (23) and dependencies ${\left[{{(\Phi }_{S})}_{L}\right]}_{E=const}=f({∆\pi }_{s}/C_{s})$ and ${\left[{{(\Phi }_{S})}_{L}\right]}_{{∆\pi }_{s}/C_{s}=const}=f(E)$ presented in Figure 10a,b, the dependencies ${\left[{{(\Phi }_{F})}_{L}\right]}_{E=const}=f({∆\pi }_{s}/C_{s})$ and ${\left[{{(\Phi }_{F})}_{L}\right]}_{{∆\pi }_{s}/C_{s}=const}=f(E)$ ${\left[{{(\Phi }_{U})}_{L}\right]}_{E=const}=f({∆\pi }_{s}/C_{s})$ and ${\left[{{(\Phi }_{U})}_{L}\right]}_{{∆\pi }_{s}/C_{s}=const}=f(E)$ were calculated. The results of the calculations are presented in Figure 11a–d. Comparing the data for ${\left[{{(\Phi }_{F})}_{L}\right]}_{E=const}=f({∆\pi }_{s}/C_{s})$ and ${\left[{{(\Phi }_{U})}_{L}\right]}_{E=const}=f({∆\pi }_{s}/C_{s})$ shown in Figure 11a,c and the data for ${\left[{{(\Phi }_{F})}_{L}\right]}_{{∆\pi }_{s}/C_{s}=const}=f(E)$ and ${\left[{{(\Phi }_{F})}_{L}\right]}_{{∆\pi }_{s}/C_{s}=const}=f(E)$ shown in Figure 11b,d, it can be seen that $\left[{{(\Phi }_{F})}_{L}\right]$ ≈ 0.01$\left[{{(\Phi }_{U})}_{L}\right]$.

Particularly important from our point of view is the analysis of energy fluxes in membrane transport processes, i.e., internal energy ${(\Phi }_{U}$, Figure 11c,d), useful energy ${(\Phi }_{F}$, Figure 11a,b) (used directly in the transport of ions) and energy dissipated in the environment ${(\Phi }_{S}$, Figure 10a,b) as a function of the thermodynamic forces applied on the membrane. In all cases of these energy fluxes, we observed slower increases with increasing thermodynamic force in the range of small force values (up to about 6 kJ/mol for concentration difference and up to about 0.9 V for electrical force). Further increasing thermodynamic forces in the tested concentration range causes faster increases in each of the considered energy fluxes without observing a clear “saturation effect”.

## 4. Discussion

All of the calculated flux–force coupling coefficients ($L_{ij}$) depend nonlinearly on thermodynamic forces Δπ/C_s_ and E. The values of these coefficients are positive over the entire range of considered solution concentrations. A positive value of the $L_{ij}$ coefficient means that an increase in the j-th thermodynamic force causes an increase in the corresponding i-th flux. Nonlinear variations of the $L_{ij}$ coefficient make the force–flux relationship more complex. The greater the slope of characteristics $L_{ij}=f({∆\pi }_{s}/C_{s},E=const.)$ and $L_{ij}=f(E,$ ${∆\pi }_{s}/C_{s}=const.$) (i, j ∈ {1, 2}), the greater the effect of a given stimulus on the corresponding flux. SEM analysis of the membrane allows us to conclude that the nonlinearities of these characteristics may be related to the structure of the membrane itself and its interaction with the individual transported substances and, thus, indirectly related to the interaction of the transported substances in the membrane. The coupling coefficients ($l_{ij}$) between the individual processes in the membrane take values ranging from zero (no coupling) to one (complete coupling). According to results of calculations, increasing both thermodynamic forces (E and ${∆\pi }_{s}/C_{s}$) causes increases in coupling between transport processes of ions in the Ultra Flo 145 Dialyser membrane. An increase in one of these thermodynamic forces causes an increase in the energy conversion efficiency coefficient, as well as increases in fluxes of free energy and dissipated energy for the Ultra Flo 145 Dialyser membrane during membrane transport of ions.

## 5. Conclusions

- The membrane transport of ions through an Ultra Flo 145 Dialyser membrane requires the extension of a linear model of membrane transport, for example, by forcing the dependence of the model’s transport coefficients on thermodynamic forces.
- Increases in thermodynamic forces (E or ${∆\pi }_{s}/C_{s}$) on the Ultra Flo 145 Dialyser membrane in the new model cause increases in all coefficients characterizing the membrane transport processes of ions and increases in energy conversion efficiency of membrane transport processes.
- Fluxes of free energy and dissipated energy also nonlinearly depend on the thermodynamic forces (E and ${∆\pi }_{s}/C_{s}$) used in the Ultra Flo 145 Dialyser membrane system with aqueous electrolytes solutions.

## Data Availability

The datasets used for this study are available upon request from the corresponding author.
